# Association of Psychopathology Symptoms, Self-Compassion, and Forgiveness in Patients With Pulmonary Embolism

**DOI:** 10.7759/cureus.19951

**Published:** 2021-11-27

**Authors:** Foteini Malli, Ioannis C Lampropoulos, Giorgos Iatrou, Ourania S Kotsiou, Fotini Bardaka, Evangelia Kotrotsiou, Evangelos C Fradelos, Konstantinos Gourgoulianis, Zoe Daniil

**Affiliations:** 1 Faculty of Nursing/Respiratory Disorders Lab, University of Thessaly, Larissa, GRC; 2 Respiratory Medicine Department, University of Thessaly, Larissa, GRC; 3 Respiratoty Medicine Department, University of Thessaly, Larissa, GRC; 4 General Department, University of Thessaly, Larissa, GRC; 5 Faculty of Nursing, University of Thessaly, Larissa, GRC; 6 Faculty of Medicine/Respiratory Medicine Department, University of Thessaly, Larissa, GRC

**Keywords:** mental health, forgiveness, self-compassion, scl-90-r, venous thromboembolism, pulmonary embolism

## Abstract

Background

Pulmonary embolism (PE) is a potentially life-threatening disease with both physical and psychological impacts. The psychological distress in the early phase of the disease has not been previously studied in the literature.

Methods

The study sample included patients with PE with or without deep vein thrombosis. All subjects included in the study prospectively completed the Symptom Checklist-90-R (SCL-90-R) questionnaire, the Heartland Forgiveness Scale (HFS), and the Self-Compassion Scale (SCS) during their hospitalization for PE.

Results

Forty-four PE patients were included in the study (59.1% males). The mean age was 62.27±15.03 years. The majority (77.3%) had at least one comorbidity with 9.1% previously diagnosed with depression. The Total Global Severity Index (GSI) score for SCL-90-R was 82.42±49.70 while 36.4% of subjects had a high “Obsessive-compulsive” score, 22.7% had a high “Depression” score, and 22.7% presented a high “Hostility” score. The total HFS score was 45.54±40.42 with 54.5% of patients classified as “usually forgiving.” The mean SCS score was 2.05±0.65 with 59.1% of patients presenting moderate self-compassion while 18.2% had low self-compassion. The total SCS score was correlated with the total GSI score (p=0.005, r=-0.576) and total HFS score (p=0.005, r=0.675). The SCS Self-kindness score correlated with interpersonal sensitivity (p=0.024, r=-0.479), depression (p=0.008, r=-0.551), and GSI score (p=0.049, r=-0.425). Self-judgement correlated with paranoid ideation (p=0.044, r=-0.467), hostility (p=0.007, r=-0.597), and GSI (p=0.027, r=-0.505). Isolation correlated with interpersonal sensitivity (p=0.026, r=-0.509), anxiety (p=0.014, r=-0.553), hostility (p=0.032, r=-0.494), paranoid ideation (p=0.026, r=-0.509), and GSI (p=0.015, r=-0.548). The total SCS score correlated with anxiety (p=0.041, r=-0.438). SCS Self-kindness score correlated significantly with total HFS score (p=0.002, r=0.613), forgiveness of self (p=0.011, r=0.528), forgiveness of others (p=0.008, r=0.550), and forgiveness of situations (p=0.004. r=0.587). Common humanity was significantly correlated with total HFS score (p=0.023, r=0.481), forgiveness of others (p=0.033, r=0.456), and forgiveness of situations (p=0.016, r=0.507). Mindfulness was positively correlated with HFS total score (p=0.009, r=0.544), forgiveness of self (p=0.049, r=0.424), forgiveness of others (p=0.012, r=0.525), and forgiveness of situations (p=0.013, r=0.520).

Conclusions

We report for the first time that patients acutely hospitalized for PE present symptoms of obsessive-compulsive disorder, depression, and hostility and exhibit moderate self-compassion. The marginal majority of PE patients are “usually forgiving” during the acute phase of the disease. Self-compassion is positively associated with forgiveness and negatively associated with psychiatric symptoms. Further studies are warranted in order to assess longitudinal differences in psychometric scores and the possible result of targeted mental health interventions at PE-specific clinical outcomes.

## Introduction

Venous thromboembolism (VTE) includes two disease entities, pulmonary embolism (PE), and deep vein thrombosis (DVT) [1]. VTE is a common disorder with an incidence of approximately 100-200 per 100,000 population [2]. PE stands as the most serious VTE complication that can be lethal in 30% if left untreated, which is reduced to 8% if diagnosed [3]. Patients with PE may experience distressing symptoms, such as syncope and/or hemoptysis, whereas the acute PE event may carry significant morbidity with right heart dysfunction, respiratory failure, and hemodynamic instability [1]. Additionally, treatment with anticoagulants for PE may result in hemorrhage that may cause further discomfort for the newly diagnosed patient.

Although PE may result in both physical and psychological distress, the latter has not been extensively studied in the literature. PE is a potentially lethal diagnosis that results in hospitalization, it may be related to acute deconditioning, may require reperfusion therapy (i.e. thrombolysis), and exerts a risk for chronic complications (i.e. adverse events, such as bleeding, chronic thromboembolic pulmonary hypertension, etc.), and recurrences [4]. The acute diagnosis can be stressful, and the patient may experience impaired quality of life, anxiety, and depression in long-term follow-up [5-6]. Psychological distress may further worsen existing illnesses and thus contribute to a decreased drive for engaging in disease management behaviors [7]. Limited data are available on the short-term psychological response to PE diagnosis, with most studies focusing on the extended impact of the disease on quality of life and psychosocial responses.

A large number of psychiatric symptoms are present in acute medical patients suffering from potentially lethal diseases [8]. The psychological characteristics may relate to physical symptoms and influence how patients manage their disease. For example, the presence of psychotic symptoms has been related to poor drug adherence [8] while self-compassion is associated with self-management behaviors [7]. The term self-compassion as described by Neff [9] includes the idea that one should treat himself/herself as he/she would treat others and is defined by three components: common humanity versus isolation, self-kindness versus self-judgment, and mindfulness versus over-identification. Self-compassion is related to improved quality of life, protects against anxiety and depression, and is associated with better clinical outcomes [7]. Forgiveness is a complex phenomenon that is considered to be central to human wellbeing and linked to health promotion by decreasing anxiety and depression. Low forgiveness scores have been associated with post-traumatic stress disorder [10].

To our knowledge, there are no published data regarding the psychological state of patients in the acute phase of PE. In the present study, we aimed to assess psychopathology symptoms, self-compassion, and forgiveness among patients with PE during their hospitalization for the acute event. To this context, we administered the Symptom Checklist-90-R (SCL-90-R), the Heartland Forgiveness Scale (HFS), and the Self-Compassion Scale (SCS) in patients experiencing acute PE.

## Materials and methods

Study population

Patients with PE with or without DVT were enrolled between February 2020 and June 2021 during their hospitalization for an acute PE at the respiratory medicine department of the University Hospital of Larissa, Greece. All patients had a PE diagnosis confirmed by computed tomography pulmonary angiography with evidence of filling defect within a vessel [11]. Concurrent DVT was diagnosed with the application of whole leg compression ultrasonography. The patients’ demographic and clinical characteristics at presentation were recorded. The patients’ comorbidities, risk factors for VTE, and adverse events (i.e. bleeding) were assessed at the patients’ follow-up at the pulmonary embolism outpatient clinic of the respiratory medicine department of the University Hospital of Larissa. Unprovoked PE was defined according to the International Society of Thrombosis and Hemostasis guidelines as a PE occurring in the absence of transient or persistent major or intermediate risk factors [1,12]. Hospitalization duration was calculated from the date of admission to the date of discharge. Patients with evidence of acute right heart dysfunction and/or high-risk PE were diagnosed as stated in recently published guidelines [1]. All subjects included in the study completed the SCL-90-R questionnaire, the HFS, and the SCS one to two days prior to their hospital discharge. The study was approved by the University Hospital of Larissa ethics committee (4589-31/1/2020). Written informed consent from all subjects was obtained.

Symptom Checklist-90-R (SCL-90-R)

SCL-90-R is a brief self-report psychometric instrument that aims to address a broad spectrum of psychological problems and symptoms of psychopathology [13] It consists of 90 items and results in nine scores of primary symptoms (somatization, obsessive-compulsive, interpersonal sensitivity, depression, anxiety, hostility, phobic anxiety, paranoid ideation, psychoticism), and three scores of global distress indices (Global Severity Index (GSI), Positive Symptom Distress Index, Positive Symptom Total). The answers are based on a five-point Likert type evaluation, ranging from “not at all” (0) to “extremely” (4) [14]. A large number of researches have been conducted concerning the SCL-90-R reliability, validity, and usefulness in everyday clinical practice, rendering it as one of the most widely used measures of distress [15]. The Greek adaptation was performed by Donias et al. [13]. Scoring of SCL-90-R was performed as previously described [14].

Heartland Forgiveness Scale (HFS)

The HFS consists of an 18-item questionnaire designed to assess a person’s dispositional forgiveness and thus one’s general tendency to be forgiving [16]. The responses follow a seven-point Likert scale (1 = almost always false for me, to 7 = almost always true for me). HFS reports on three subscales (Forgiveness of Self, Forgiveness of Others, Forgiveness of Situations) and the total scale. A higher score corresponds to an individual’s greater willingness for forgiveness towards others, himself (or herself), and/or situations [17]. A total score of 18 to 54 indicates that one is usually unforgiving, a total score of 55 to 89 suggests that a person is as likely to forgive, as he/she is not to forgive and a total score of 90 to 126 reflects that one is usually forgiving.

Self-Compassion Scale (SCS)

The SCS is a 26-item self-report questionnaire that addresses self-compassion [9]. The instrument results in three subscales with a positive direction (Self-Kindness, Common Humanity, Mindfulness), and three subscales with a negative direction (Self-Judgment, Isolation, Over-Identification). The responses follow a five-point Likert scale, ranging from 1 (very rarely) to 5 (very often). Subjects with higher scores present higher levels of self-compassion. Values of 1-2.5 indicate low self-compassion, 2.5-3.5 indicate moderate, and 3.5-5.0 indicate high self-compassion [18]. The present study used the Greek validated version of the SCS [19].

Statistical analysis

Data are presented as mean ± standard deviations (SD) or as percentages. Normal distribution was assessed by the Kolmogorov-Smirnov test. Univariate correlations between continuous demographic characteristics (i.e. age) and the results of the instruments (SCL-90-R, SCS, Self-Forgiveness Scale) were performed by Pearson’s correlation coefficient for variables that were normally distributed or by Spearman’s correlation coefficient for variables that were not normally distributed. The chi-square test or Fischer’s exact test was used for the univariate analysis of qualitative variables (i.e., gender, comorbidities, presence of symptoms) and student’s t-test or Mann-Whitney U test for quantitative variables (i.e., age, scores of the instruments) according to variable distribution. The one-way analysis of variance (ANOVA) test of repeated measures or the Kruskal Wallis test was applied to investigate whether there are any statistically significant differences between the means of three or more independent groups (i.e., number of comorbidities) according to the variable distribution. The reliability was tested with Cronbach’s alpha estimator, with a range of 0 to 1. Values higher than 0.7 indicate good internal consistency of the items. A p-value of <0.05 was considered to be statistically significant. Analysis was performed using the SPSS 20 statistical package (IBM Corp., Armonk, NY).

## Results

Patient characteristics

**Figure 1:**
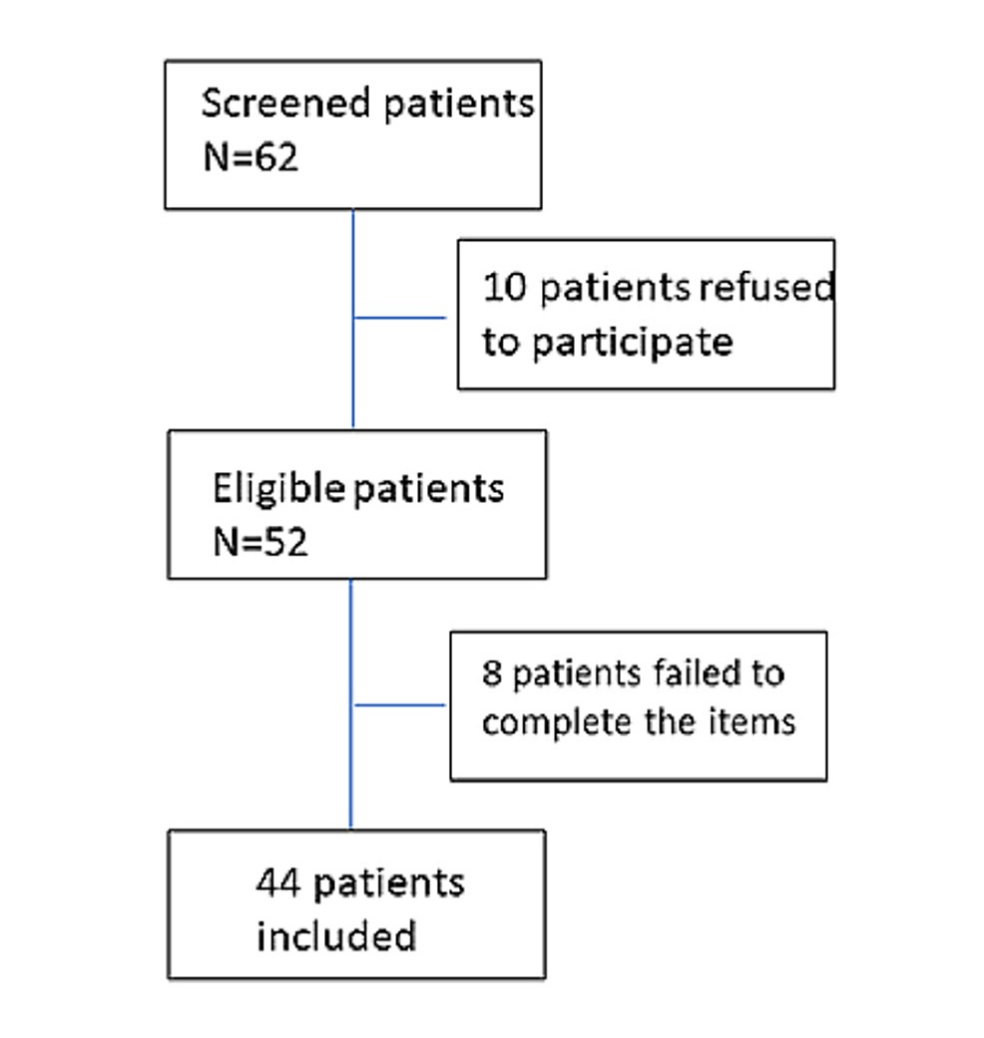
Flow diagram for study participants

Forty-four patients with recently diagnosed PE were included in the present study with 22.7% presenting concurrent DVT. The study flow diagram for study participants is presented in Figure 1. The mean age was 62.27±15.03 years and the majority (59.1%) were males. Most of the patients (68.2%) were never or ex-smokers and 31.8% were never smokers. The majority (77.3%) had at least one comorbidity. Arterial hypertension was present in 13.6% of the patients and 13.6% had diabetes mellitus. Bronchial asthma was present in 9.1% of the population, 9.1% had thyroid disease, 4.5% had bronchiectasis, and 4.5% of the patients had a history of coronary heart disease. The majority (68.2%) of patients presented with thoracic pain, 31.8% claimed shortness of breath, 18.2% hemoptysis, and 9.1% of patients experienced syncope. Incidental PE was evident in 9.1% of patients. As far as PE severity is concerned, 13.6% of the patients had signs of right heart dysfunction in echocardiographic assessment at admission and 9.1% had increased troponin values. None had high-risk PE requiring reperfusion therapy. The clinical and demographic characteristics of our cohort are summarized in Table 1.

**Table 1:** Demographic and clinical characteristics of the study population

Characteristic	Mean ±SD or %
Age (years)	62.27±15.03
Gender Male	59.1%
Female	40.9%
Comorbidities None	18.2%
≥1	81.8%
Symptoms	
Thoracic pain	68.2%
Dyspnea	31.8%
Fever	18.2%
Hemoptysis	18.2%
Syncope	9.1%
Leg swelling	9.1%
Heart palpations	4.5%
None (incidental PE)	9.1%

Only 4.5% of the subjects had a prior VTE history and 4.5% had a positive family history of VTE. Of the patients included, 18.2% had been subjected to surgery recently (within the previous 30 days), 4.5% had cancer-associated thrombosis, 4.5% had chronic respiratory failure requiring long-term oxygen therapy, and 9.1% had a history of stroke with reduced mobility. Mean hospitalization days were 7.92±5.59 days. None of the patients included had pregnancy or coronavirus disease (COVID)-associated PE. Of the patients included in the study, 9.1% were previously diagnosed with depression. Unprovoked PE was present in 45.5% of the study population. Only 4.5% of the patients presented minor bleeding following the initiation of anticoagulation and none reported clinically relevant non-major bleeding or major bleeding events. As far as hereditary thrombophilia is concerned, 9.1% of the population were heterozygous for FV Leiden and 9.1% were heterozygous for prothrombin G20210A mutation. None of the patients included had antithrombin, protein S, or protein C deficiency.

SCL-90-R

The descriptive statistics SCL-90-R are presented in Table 2. The total GSI score for SCL-90-R was 82.42±49.70. The majority of patients included were characterized by normal scores on the nine subscales of the SCL-90-R. Briefly, 36.4% of subjects had a high obsessive-compulsive score, 22.7% had a high depression score, 22.7% presented high hostility score, 18.2% exhibited a high-level score on the interpersonal sensitivity scale, 18.2% had a high level of paranoid ideation score, 13.6% of subjects had a high somatization score, 13.6% had increased level of anxiety scale, 9.1% had increased phobic anxiety scale, and 4.5% exhibited increased score on the psychotism scale. Cronbach's alpha value was 0.832.

**Table 2:** Results of SCL-90-R, HFS, and SCS scale SCL-90-R: Symptom Checklist-90-R; HFS: Heartland Forgiveness Scale; SCS: Self-Compassion Scale

Variable	Mean ±SD
SCL-90-R	
Somatization	1.14±0.35
Obsessive-compulsive	1.36±0.49
Interpersonal sensitivity	1.18±0.34
Depression	1.23±0.43
Anxiety	1.14±0.35
Hostility	1.32±0.57
Phobic anxiety	1.18±0.50
Paranoid ideation	1.18±0.39
Psychotism	1.05±0.21
Total GSI score	82.42±49.70
Self-compassion scale	
Self-kindness	1.82±0.85
Common humanity	1.59±0.85
Mindfulness	1.95±0.84
Self-judgment	2.16±0.76
Isolation	2.16±0.68
Over identification	2.11±0.87
Total SCS score	2.05±0.65
Heartland forgiveness scale	
Forgiveness of self	14.95±14.29
Forgiveness of others	16.22±14.67
Forgiveness of situations	14.36±14.53
Total HFS score	45.54±40.42

Heartland Forgiveness Scale

The total HFS score was 45.54±40.42 (Table 2). Of the patients included, 54.5% were classified as “usually unforgiving,” 27.3% were categorized as “as likely to forgive, as he/she is not to forgive,” and 18.2% were classified as “usually unforgiving.” As far as the subscales of HFS are concerned, the forgiveness of self score was 14.95±14.29, the forgiveness of others score was 16.22±14.67, and the forgiveness of situations score was 14.36±14.53. Cronbach's alpha value was 0.871.

Self-Compassion Scale

The mean SCS score was 2.05±0.65 (Table 2). The majority of the patients presented moderate self-compassion score (59.1%), 22.7% demonstrated high self-compassion score, and 18.2% had a low self-compassion score. The SCS subscales were in the low self-compassion range. Namely, the self-kindness score was1.82±0.85, common humanity score was 1.59±0.85, mindfulness score was 1.95±0.84, self-judgment score was 2.16±0.76, isolation score was 2.16±0.68, and over-identification score was 2.11±0.87. Cronbach's alpha value was 0.763.

Bivariate correlations

We examined for significant correlations between the SCL-90-R, SCS, and HFS scores (Table 3). The SCL-90-R subscales displayed significant intercorrelations. In more detail, the SCL-90-R somatization score was significantly correlated with the obsessive-compulsive score (p<0.001, r=0.823), interpersonal sensitivity score (p<0.001, r=0.789), depression score (p=0.002, r=0.625), anxiety score (p<0.001, r=0.740), hostility score (p=0.001, r=0.673), phobic anxiety score (p<0.001, r=0.797), and paranoid ideation score (p=0.011, r=0.533). The obsessive-compulsive score was significantly correlated with the interpersonal sensitivity score (p<0.001, r=0.849), depression score (p<0.001, r=0.836), anxiety score (p<0.001, r=0.736), hostility score (p<0.001, r=0.769), phobic anxiety score (p<0.001, r=0.820), psychotism score (p=0.006, r=0.571), and paranoid ideation score (p=0.005, r=0.575). The interpersonal sensitivity score was significantly correlated with the depression score (p<0.001, r=0.873), anxiety score (p=0.001, r=0.638), hostility score (p<0.001, r=0.696), phobic anxiety score (p<0.001, r=0.895), and paranoid ideation score (p=0.001, r=0.664). The depression score was significantly correlated with the anxiety score (p=0.002, r=0.612), hostility score (p=0.002, r=0.627), phobic anxiety score (p<0.0001, r=0.692), and paranoid ideation score (p<0.001, r=0.686). The anxiety score was significantly correlated with the hostility score (p<0.001, r=0.773), phobic anxiety score (p<0.001, r=0.734), psychotism score (p=0.001, r=0.645), and paranoid ideation score (p=0.008, r=0.549). The phobic anxiety score was significantly correlated with the psychotism score (p=0.002, r=0.617) and Paranoid ideation score (p=0.011, r=0.533). The psychotism score was significantly correlated with the paranoid ideation score (p=0.026, r=0.473).

**Table 3:** Bivariate intercorrelations of the SCL-90-R, SCS, and HFS subscales SCL-90-R: Symptom Checklist-90-R; HFS: Heartland Forgiveness Scale; SCS: Self-Compassion Scale

Variables	SCL-90-R									
	Somatization	Obsessive-compulsive	Interpersonal sensitivity	Depression	Anxiety	Hostility		Phobic anxiety	Paranoid ideation	Psychotism
Somatization	Not applicable	p=<0.001, r=0.823	p=<0.001, r=0.789	p=0.002, r=0.625	p<0.001, r=0.740	p=0.001, r=0.673		p<0.001, r=0.797	p=0.001, r=0.533	p>0.05
Obsessive-compulsive	p=<0.001, r=0.823	Not applicable	p<0.001, r=0.849	p<0.001, r=0.836	p<0.001, r=0.736	p<0.001, r=0.769		p<0.001, r=0.820	p=0.005, r=0.575	p=0.006, r=0.571
Interpersonal sensitivity	p=<0.001, r=0.789	p<0.001, r=0.849	Not applicable	p<0.001, r=0.873	p=0.001, r=0.638	p<0.001, r=0.696		p<0.001, r=0.895	p=0.001, r=0.664	p>0.05
Depression	p=0.002, r=0.625	p<0.001, r=0.836	p<0.001, r=0.873	Not applicable	p=0.002, r=0.612	p=0.002, r=0.627		p<0.001, r=0.692	p<0.001, r=0.686	p>0.05
Anxiety	p<0.001, r=0.740	p<0.001, r=0.736	p=0.001, r=0.638	p=0.002, r=0.612	Not applicable	p<0.001, r=0.773		p<0.001, r=0.734	p=0.008, r=0.549	p=0.001, r=0.645
Hostility	p=0.001 r=0.673	p<0.001, r=0.769	p<0.001, r=0.696	p=0.002, r=0.627	p<0.001, r=0.773	Not applicable		p>0.05	p>0.05	p>0.05
Phobic anxiety	p<0.001, r=0.797	p<0.001, r=0.820	p<0.001, r=0.895	p<0.001, r=0.692	p<0.001, r=0.734	p>0.05		Not applicable	p=0.011, r=0.533	p=0.002, r=0.617
Paranoid ideation	p=0.001, r=0.533	p=0.005, r=0.575	p=0.001, r=0.664	p<0.001, r=0.686	p=0.008, r=0.549	p>0.05		p=0.011, r=0.533	Not applicable	p=0.026, r=0.473
Psychotism	p>0.05	p=0.006, r=0.571	p>0.05	p>0.05	p=0.001, r=0.645	p>0.05		p=0.002, r=0.617	p=0.026, r=0.473	Not applicable
	SCS						HFS			
	Self-kindness	Common humanity	Mindfulness	Self-judgment	Isolation	Over-identification			Forgiveness of self	Forgiveness of situations
Self-kindness	Not applicable	p>0.05	p=0.002, r=0.656	p>0.05	p>0.05	p>0.05		Forgiveness of self	Not applicable	p=0.001, r=0.642
Common humanity	p>0.05	Not applicable	p=0.046, r=0.656	p>0.05	p>0.05	p>0.05		Forgiveness of others	P<0.001, r=0.821	P<0.001, r=0.902
Mindfulness	p=0.002, r=0.656	p=0.046 r=0.656	Not applicable	p>0.05	p>0.05	p>0.05		Forgiveness of situations	p=0.001, r=0.642	Not applicable
Self judgement	p>0.05	p>0.05	p>0.05	Not applicable	p=0.024 r=0.516	p<0.001, r=0.761				
Isolation	p>0.05	p>0.05	p>0.05	p=0.024, r=0.516	Not applicable	p=0.004, r=0.626				
Over identification	p>0.05	p>0.05	p>0.05	p<0.001, r=0.761	p=0.004, r=0.626	Not applicable				

In the same context, SCS subscales displayed statistically significant intercorrelations. Namely, the self-kindness score was significantly correlated with the mindfulness score (p=0.002, r=0.656), common humanity was significantly correlated with the mindfulness score (p=0.046, r=0.430), self-judgment was significantly correlated with over-identification (p<0.001, r=0.761), and the isolation score (p=0.024, r=0.516) and over-identification was significantly correlated with isolation (p=0.004, r=0.626). Similarly, HFS subscales demonstrated significant intercorrelations as follows. Forgiveness of self is significantly correlated with forgiveness of others (p<0.001, r=0.821) and forgiveness of situations (p=0.001, r=0.642) while forgiveness of others was significantly correlated with forgiveness of situations (p<0.001, r=0.902).

We assessed for significant correlations between the instruments used in the present study (Table 4). Total SCS score was significantly negatively correlated with the total GSI score (p=0.005, r=-0.576) and positively associated with the total HFS score (p=0.005, r=0.675). We observed significant negative correlations between the SCS subscales and SCL-90-R subscales. In more detail, the SCS self-kindness score correlated with interpersonal sensitivity (p=0.024, r=-0.479), depression (p=0.008, r=-0.551), and GSI score (p=0.049, r=-0.425). Self-judgement correlated with paranoid ideation (p=0.044, r=-0.467), hostility (p=0.007, r=-0.597), and GSI (p=0.027, r=-0.505). Isolation correlated with Interpersonal sensitivity (p=0.026, r=-0.509), anxiety (p=0.014, r=-0.553), hostility (p=0.032, r=-0.494), paranoid ideation (p=0.026, r=-0.509), and GSI (p=0.015, r=-0.548). The total SCS score correlated with anxiety (p=0.041, r=-0.438).

**Table 4:** Bivariate correlations between the SCS, SCL-90-R, and HFS subscales SCL-90-R: Symptom Checklist-90-R; HFS: Heartland Forgiveness Scale; SCS: Self-Compassion Scale

Variables	SCS					
	Total SCS score	Self-kindness score	Common humanity score	Mindfulness score	Self-judgment score	Isolation score
SCL-90-R						
Total GSI Score	p=0.005, r=-0.576	p=0.049, r=-0.425	p>0.05	p>0.05	p=0.027, r=-0.505	p=0.015, r=-0.548
Interpersonal sensitivity score	p>0.05	p=0.024, r=-0.479	p>0.05	p>0.05	p>0.05	p=0.026, r=-0.509
Depression score	p>0.05	p=0.008, r=-0.551	p>0.05	p>0.05	p>0.05	p>0.05
Hostility score	p>0.05	p>0.05	p>0.05	p>0.05	p=0.007, r=-0.597	p=0.032, r=-0.494
Paranoid ideation score	p>0.05	p>0.05	p>0.05	p>0.05	p=0.044, r=-0.467	p=0.026, r=-0.509
Anxiety score	p=0.041, r=-0.438	p>0.05	p>0.05	p>0.05	p>0.05	p=0.014, r=-0.553
HFS						
HFS total score	p=0.005, r=0.675	p=0.002, r=0.613	p=0.023, r=0.481	p=0.009, r=0.544	p>0.05	p>0.05
Forgiveness of self score	p>0.05	p=0.011, r=0.528	p>0.05	p=0.049, r=0.424	p>0.05	p>0.05
Forgiveness of others score	p>0.05	p=0.008, r=0.550	p=0.033, r=0.456	p=0.012, r=0.525	p>0.05	p>0.05
Forgiveness of situations score	p>0.05	p=0.004, r=0.587	p=0.016, r=0.507	p=0.013, r=0.520	p>0.05	p>0.05

Significant correlations were observed between the SCS and Forgiveness subscales. The SCS self-kindness score correlated significantly with the total HFS score (p=0.002, r=0.613), forgiveness of self (p=0.011, r=0.528), forgiveness of others (p=0.008, r=0.550), and forgiveness of situations (p=0.004. r=0.587). Common humanity was significantly associated with total HFS score (p=0.023, r=0.481), forgiveness of others (p=0.033, r=0.456), and forgiveness of situations (p=0.016, r=0.507), whereas we observed a trend towards a positive correlation with forgiveness of others that was not statistically significant (p=0.052, r=0.420). Mindfulness was positively correlated with HFS total score (p=0.009, r=0.544), forgiveness of self (p=0.049, r=0.424), forgiveness of others (p=0.012, r=0.525), and forgiveness of situations (p=0.013, r=0.520). We did not observe significant correlations of HFS total score or its subscales with the negative subscales of the SCS.

Comparison between patient characteristics and psychometric scores

We examined for possible significant differences of the scales according to patient characteristics. Age correlated significantly with the total HFS score (p=0.025, r=0.476), forgiveness of self score (p=0.022, r=0.485), SCS mindfulness (p=0.035, r=0.451), and over-identification (p=0.033, r-=0.490). As far as symptoms are concerned, patients reporting hemoptysis when compared to patients without hemoptysis had a higher total HFS score (82.25±11.44 vs 33.05±36.74, respectively, p<0.001) and a higher total SCS score (3.39±0.33 vs 2.48±1.25, respectively, p=0.033). We did not observe significant differences when patients were classified according to their symptoms (besides hemoptysis, which is presented earlier), comorbidities, indices of disease severity, and predisposing factors of VTE.

## Discussion

To our knowledge, this is the first study in the literature assessing psychiatric symptoms, forgiveness, and self-compassion in newly diagnosed patients with PE. We observed that a significant proportion of patients presented symptoms of obsessive-compulsive disorder, depression, and hostility, while self-compassion was in the moderate range in most of the patients. The marginal majority of our cohort were classified as “usually forgiving,” whereas forgiveness was significantly associated with self-compassion. Additionally, we observed significant negative correlations between the SCS subscales and psychopathology indices.

Studies have previously suggested that medical patients suffering from acute life-threatening diseases may exhibit moderate to severe psychiatric symptoms [8]. A significant proportion (36.4%) of our cohort exhibit signs and symptoms of obsessive-compulsive disorder, 22.7% demonstrated signs of depression and hostility, and 18.2% presented high interpersonal sensitivity. Obsessive-compulsive symptoms focus on the existence of thoughts that recur continuously and irresistibly [14]. The depression scale of SCL-90-R refers to the presence of symptoms such as depressive mood, pessimism, despair, lack of motive, as well as suicidal thought. Interpersonal sensitivity includes feelings of insufficiency and weakness and the reflection of these feelings to the social relations that results in feeling uncomfortable because of these relations. Finally, hostility comprises feelings of anger, restlessness, hostility, jealousy, and aggression. Understanding the psychological symptoms of patients with PE may facilitate the clinical management of the disease and underline the potential importance of the availability of psychosocial and mental health services for inpatients suffering from the disease.

Most of our patients presented moderate self-compassion. Self-compassion has emerged as an important part of the management of psychological issues that may be associated with many aspects of mental wellbeing, including reduced anxiety, stress, and depression, as well as improved quality of life [7]. In our cohort, the SCS score was statistically significantly negatively associated with the SCL-90-R total GSI score, thus providing further support to the previously mentioned concept that low self-compassion may be adversely related to psychological health. Published data suggest a positive association of self-compassion to medical disease management (i.e., with adherence to behaviors aiming at health promotion) [20]. Additionally, self-compassion aimed interventions (such as compassion-focused therapy or compassionate mind training) in patients with chronic conditions may improve health-promoting behaviors and affect clinical outcomes [7]. The design of our study cannot assess the possible association between the mental distress associated with acute PE and the patient’s response to disease management. The long-term results of self-compassion targeted therapies in patients experiencing PE merits attention.

The marginal majority (54.5%) of our patients were classified as “usually forgiving,” with the rest classified as either ‘“as likely to forgive, as he/she is not to forgive,” or as “usually unforgiving.” Forgiveness presents an adaptive emotion that focuses on coping strategies to overcome negative emotions, such as anger, anxiety, and depression while embracing a positive attitude towards life and disease [10]. Studies have shown that forgiveness is positively associated with health-related quality of life while forgivers usually tend to suppress behaviors damaging to their mental and/or physical health. Forgiveness is considered a powerful tool with significant health impacts on the forgiver in both physical and psychological aspects of health [21]. Physiological changes associated with forgiveness (that could be helpful in the management of PE patients) include reduced heart rate and blood pressure and improved cardiovascular recovery [22]. Through its biological benefits, forgiveness interventions may have a significant personal health impact in patients with PE. Future studies are warranted in order to examine this hypothesis.

We observed a significant positive correlation of forgiveness with self-compassion. The positive correlation was more evident between the positive subscales of SCS (self-kindness, common humanity, and mindfulness) and the HFS total score and subscales. Previous studies have identified that self-compassion moderates the association of forgiveness and depression in the general population [23] while self-compassion may reduce self-punitiveness and increase self-forgiveness [24]. Our results are in accordance with the aforementioned findings and provide further insight into the relationship between self-compassion and forgiveness in PE patients.

The most common symptoms in our cohort were thoracis pain and dyspnea followed by fever, hemoptysis, syncope, and leg swelling. The distribution of symptoms in our cohort does not differ from those previously reported in the literature. Others have previously reported that thoracic pain and dyspnea are the most frequent presenting symptoms while syncope may be present in 6%-39% according to the cohort studied [25].

Studies have previously examined the mental health of patients with PE during their follow-up. PE is now considered a disease requiring long-term follow-up since patients may experience both physical as well as mental consequences of the disease. Noble et al. have demonstrated that patients with PE may have features of post-traumatic stress disorder and anxiety [6]. Additionally, the majority of patients may display impaired quality of life during their follow-up [5]. Further studies are warranted in order to clarify the mental effect of PE, both acutely and in the long term.

We acknowledge that our study is not without limitations. We have included a small sample size. However, we chose to include only patients with PE with a recent diagnosis that significantly narrows the pool of patients that could participate in our study. Additionally, we did not longitudinally assess the psychometric instruments, and therefore, we cannot address potential differences in psychiatric symptoms, self-compassion, and forgiveness over time. We acknowledge that our results are limited to the acute effect of PE and cannot be extrapolated in the mental health of patients during their follow-up while the associations cannot imply causality. Additionally, our results may be implicated by the fact that the patients were assessed during the pandemic, whereas the data may be biased by the patients’ previous mental health. Lastly, the presence of comorbidities may interfere with the results of the psychometric tests. However, in real life, most PE patients suffer from comorbidities and excluding those that have other diseases would limit the extrapolation of our results in the majority of PE patients.

## Conclusions

In conclusion, we report for the first time that patients acutely hospitalized for PE present symptoms of obsessive-compulsive disorder, depression, hostility, and moderate self-compassion. The marginal majority of PE patients are “usually forgiving” during the acute phase of the disease. Self-compassion is positively associated with forgiveness and negatively associated with psychiatric symptoms. Further studies are warranted in order to assess longitudinal differences in the psychometric scores and the possible result of targeted mental health interventions on PE-specific clinical outcomes.
